# A novel strategy for driving car brain–computer interfaces: Discrimination of EEG-based visual-motor imagery

**DOI:** 10.1515/tnsci-2020-0199

**Published:** 2021-11-30

**Authors:** Zhouzhou Zhou, Anmin Gong, Qian Qian, Lei Su, Lei Zhao, Yunfa Fu

**Affiliations:** Department of Automation, Faculty of Information Engineering and Automation, Kunming University of Science and Technology, Kunming, 650500, China; Brain Cognition and Brain-computer Intelligence Integration Group, Kunming University of Science and Technology, Kunming, 650500, China; Department of Communication Engineering, School of Information Engineering, Chinese People’s Armed Police Force Engineering University, Xi’an, 710000, China; Department of Electronic Science and Applied Physics, Faculty of Science, Kunming University of Science and Technology, Kunming, 650500, China

**Keywords:** visual-motor imagery, Hilbert–Huang transformation, EEG, brain–computer interface, SVM

## Abstract

A brain–computer interface (BCI) based on kinesthetic motor imagery has a potential of becoming a groundbreaking technology in a clinical setting. However, few studies focus on a visual-motor imagery (VMI) paradigm driving BCI. The VMI-BCI feature extraction methods are yet to be explored in depth. In this study, a novel VMI-BCI paradigm is proposed to execute four VMI tasks: imagining a car moving forward, reversing, turning left, and turning right. These mental strategies can naturally control a car or robot to move forward, backward, left, and right. Electroencephalogram (EEG) data from 25 subjects were collected. After the raw EEG signal baseline was corrected, the alpha band was extracted using bandpass filtering. The artifacts were removed by independent component analysis. Then, the EEG average instantaneous energy induced by VMI (VMI-EEG) was calculated using the Hilbert–Huang transform (HHT). The autoregressive model was extracted to construct a 12-dimensional feature vector to a support vector machine suitable for small sample classification. This was classified into two-class tasks: visual imagination of driving the car forward versus reversing, driving forward versus turning left, driving forward versus turning right, reversing versus turning left, reversing versus turning right, and turning left versus turning right. The results showed that the average classification accuracy of these two-class tasks was 62.68 ± 5.08%, and the highest classification accuracy was 73.66 ± 6.80%. The study showed that EEG features of O1 and O2 electrodes in the occipital region extracted by HHT were separable for these VMI tasks.

## 1 Introduction

Brain–computer interface (BCI) neural activity learned to generate user’s perception or cognitive activity patterns for controlling external devices [1]. BCI can improve the quality of life of patients suffering from severe motor disorders. It can also communicate and control modes of healthy people. BCI based on imaginative mental activity is an important brain–computer interaction. In BCI, the traditional imagery task is motor imagery (MI) [2,3], which requires subjects to feel or recall the movement of specific parts of their body, such as hands or feet [4,5,6,7,8,9,10]. However, learning and controlling MI mental activities are difficult and often require many hours of training. This could impose a burden on subjects, thereby reducing BCI users’ acceptance. The MI-BCI performance is associated with the amount of training received by the subjects. However, the classification performance of this BCI is unreliable [4,11] owing to a serious “BCI illiteracy” [12,13,14]. This can affect the performance and application of the BCI.

Many earlier studies have achieved remarkable results on the MI mental activity (the neurological principle of brain structure and function) [15,16,17] and on analyzing and decoding the algorithm of brain signals related to MI [15]. However, BCI is limited to laboratory demonstrations, and its practical application is difficult [16]. Therefore, MI may be unsuitable in controlling BCI, especially for patients with motor disorders [15]. Using the MI method, it will be difficult to mentally rehearse the patients’ movement process.

Compared with the above-mentioned MI mental activity, visual motor imagery (VMI) is a kind of mental activity that is easy to learn and control. It requires subjects to view a specific picture or scene in their brain from a third-person perspective [4,8,9,10]. The novel aspect of VMI is that it only requires fewer hours of training [15], and people will be proficient within a few hours since they often carry out such mental activities in their daily life. In addition, VMI is a natural mental activity similar to children viewing their mother’s face and adults viewing portraits. Thus, it is easy to reflect the image of an object in our brain and to mentally manipulate the object with various movements. We can also recall a movie or a social scene in our daily life. In particular, it is easy to reflect the movements of other people’s limbs in our brain. Thus, VMI activities contrast to MI activities, which require subjects to mentally rehearse movements of specific parts of their body but not the actual execution of a movement. That is, subjects must mentally rehearse an action and at the same time prevent the action from happening [4,5,12,13,14,16]. This is a contradictory confrontational process and an unnatural mental activity. This is difficult to implement and control as people rarely carry out such mental activities in their daily life. Compared with MI, VMI is a better mental task to control BCI [15]. Therefore, this study designs a new VMI-BCI experimental paradigm to identify the VMI tasks for BCI.

Compared with the traditional MI-BCI, few studies focus on VMI-BCI [15]. Neuper et al. [18] explored kinesthetic MI, VMI, movement execution, and movement observation. Frequency band and electrode position are extracted as features and classified using the distinction sensitive learning vector quantization algorithm. From the results, the average classification accuracy of movement execution and movement observation is approximately 80%, the average classification accuracy of kinesthetic MI is approximately 67%, and the average classification accuracy of VMI is approximately 56%. Azmy and Safri [19] used BCI based on visual imagery electroencephalogram (EEG) to control a robot. In extracting the power spectrum features, the F8 electrode located in the frontal region is the best position for detecting VMI EEG, which responds to the oscillation rhythm in the alpha band. However, the classification accuracy and classifier are absent. Sousa et al. [20] classified three visual imagery tasks of static points, dynamic points moving vertically in two directions, and dynamic points moving in four directions of up, down, left, and right. The power spectrum energy feature is extracted and classified by a support vector machine (SVM) with an average classification accuracy of 87.64%. Kosmyna et al. [15] classified the visual imagination of predefined flowers and hammers. The feature of the power spectrum is extracted and classified by spectral weighed common spatial patterns. From the results, the classification accuracy is 52% of the visual imagination flower versus the visual imagination hammer. These studies on VMI-BCI limit the extracted features to the power spectrum, and it is necessary to introduce a new feature extraction method.

Traditional EEG feature extraction methods are autoregressive (AR) model, adaptive model, wavelet transform, and common space model. The Fourier transform wavelet method uses sinusoidal components of different frequencies to fit the original signal. It is difficult for the wavelet method to achieve a high resolution in both the time domain and the frequency domain [21]. Compared with the wavelet transform, the Hilbert–Huang transform (HHT) can obtain high-resolution information simultaneously in the time domain and the frequency domain. HHT is a new signal processing method, proposed by Huang et al., suitable for nonlinear and nonstationary signal analysis [21]. EEG is a highly nonlinear and nonstationary signal. Sun et al. [22] show that EEG feature extraction for MI based on HHT is superior to the traditional wavelet transform and the feature extraction method without HHT processing. Thus, this study attempts to introduce HHT into VMI-BCI feature extraction to verify the effectiveness of the method.

The rest of the article is organized as follows. Section 2 introduces the materials and methods in designing a novel VMI-BCI experimental paradigm; Section 3 presents the results; Section 4 provides the discussions; and Section 5 concludes the article.

## 2 Materials and methods

### 2.1 Subjects

Using the kinesthetic and visual-motor imagery questionnaire (KVMIQ) [23,24,25,26,27,28,29,30,31,32,33,34,35], a total of 30 healthy subjects (numbered sub1–sub30) with strong visual imagination (question score ≥70) were recruited to participate in data collection. All subjects were male, right handed, and aged 25 ± 1 years. Their visual acuity was normal or corrected to normal, and they have no mental illness. None of the subjects had taken part in this type of experiment.


**Informed consent:** Informed consent has been obtained from all individuals included in this study.
**Ethical approval:** The research related to human use has been complied with all the relevant national regulations, institutional policies, and in accordance the tenets of the Helsinki Declaration and has been approved by the Medical Ethics Committee of Kunming University of Science and Technology.

### 2.2 Experimental paradigm, process, and settings

#### 2.2.1 Visual imagination paradigm design

**Figure 1:**
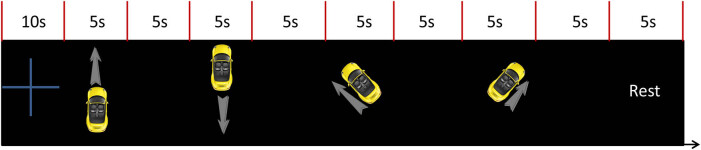
Visual imagination experimental paradigm (animated in the experiment).

To control the car or robot to move forward, move backward, turn left, and turn right naturally by mental activity, four VMI tasks were designed: imagine the car moving forward, reversing, turning left, and turning right as shown in Figure 1. The test began with a fixed cross on the screen for 10 s, prompting the subjects to start the experiment. The fixed cross disappeared, and the screen showed the animation of the car moving forward for 5 s. The subjects must observe the animation (i.e., visual observation) and pay attention to the direction of the car moving forward. When the prompted animation disappeared, the screen was empty. For 5 s, the subjects must retain the visual mental imagery of the prompted animation from a third-person perspective (i.e., visual imagination). Afterward, the screen presents an animation of the car reversing for 5 s, and the subjects were asked to observe the animation and pay attention to the car’s backward direction. After the prompted animation disappeared, the screen was blank, and the subjects were asked to perform the animation visual imagination for 5 s. By analogy, the task of visual observation versus visual imagination is prompted to turn left and right. Finally, the vehicle is prompted to rest for 5 s and complete a block test. Each participant completed 50 blocks and 50 trials for visual observation versus VMI of the car moving forward, reversing, turning left, and turning right.

#### 2.2.2 Experimental process

Subjects were allowed to have enough sleep before the experiment to maintain a good mental state. Before the experiment, the main tester, the EEG acquisition equipment, was connected, and the relevant software was launched. Subjects were seated in comfortable chairs, about 70 cm away from the visual cue screen, with their hands flat on the table. The tester wears a brain cap and injects the brain electric cream into the subjects until the electrode impedance falls to a suitable range. In the beginning, the subjects completed the visual observation and visual imagery tasks according to the prompts of Figure 1. Subjects were asked to avoid movement and blinking during the VMI tasks.

#### 2.2.3 Experimental settings

**Figure 2:**
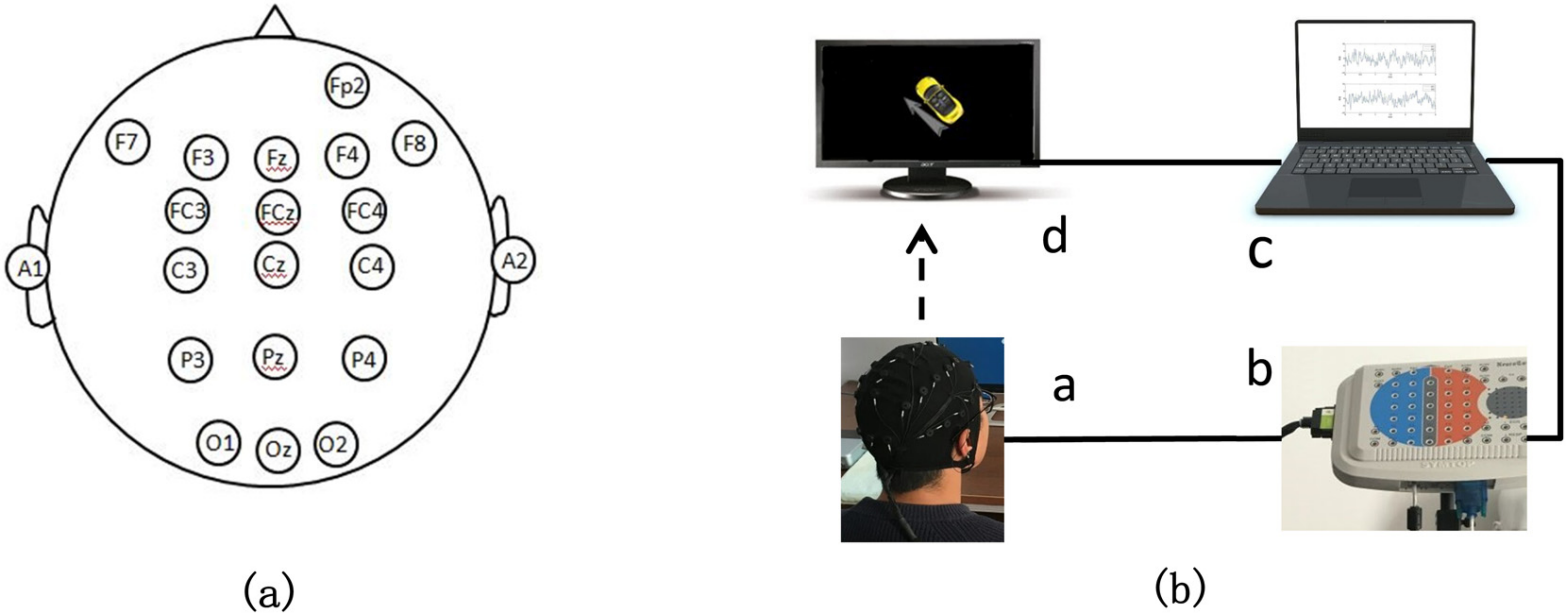
Electrode layout and experimental setup: (a) electrode layout (the reference electrodes are A1 and A2) and (b) (experimental setup (a) is the subject, (b) is the brain electrical amplifier, (c) is the data acquisition and processing computer, and (d) is the tip for visual imagination tasks).

The experimental setup is shown in Figure 2. Earlier studies [15,18,20] revealed that the VMI was correlated with the right frontal cortex and parietal cortex. In this study, the designed schematic of the electrode layout is shown in Figure 2(a). Fp2, F7, F8, F3, F4, Fc3, Fc4, C3, C4, P3, P4, O1, O2, Fz, Fcz, Cz, Pz, and Oz are 18 recording channels, and the reference electrodes are A1 and A2. Figure 2(b) shows the experimental setup. The TCL 24 inch LCD is used for the VMI task, a Lenovo ThinkPad computer with a Windows 10 operating system is used for data acquisition and processing, and MATLAB is installed on the computer for data acquisition and data processing. The EEG amplifier is the NT9200 series of Beijing Zhongke Xintuo Instrument Co., Ltd., (Beijing, China) with a sampling rate of 1,000 Hz and a 45 Hz low-pass filter.

### 2.3 Visual imagination EEG signal processing

#### 2.3.1 Data preparation

**Figure 3:**
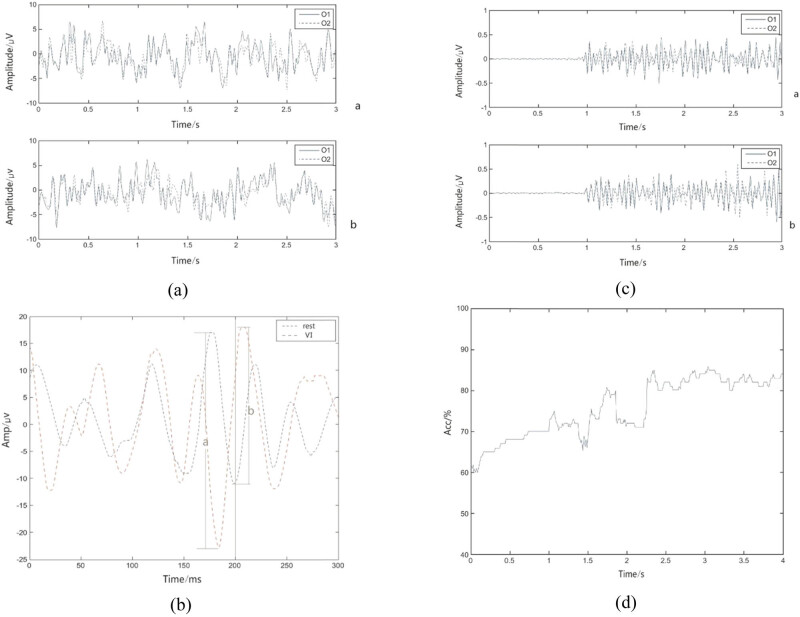
The original EEG signal of VMI, the total average waveform contrast of rest and VMI, the waveform after preprocessing, and the waveform after classification. (a) The original EEG signal of the O1 and O2 channels during visual imagery of the car forward versus reverse driving (a is the original EEG signal of the car driving forward and b is the original EEG signal of the car reverse). (b) A comparison of the total average waveform of the resting state versus the visual imagination. (c) (The waveforms of the O1 and O2 channels undergoing baseline drift correction, 8–13 Hz bandpass filtering, and ICA processing during visually imagined car forward versus reverse driving (a) is the waveform after processing when the car is driven forward and (b) is the waveform after processing when reversing)). (d) The average classification accuracy of the sub1 visually imagined car traveling forward versus reverse.

**Figure 4:**
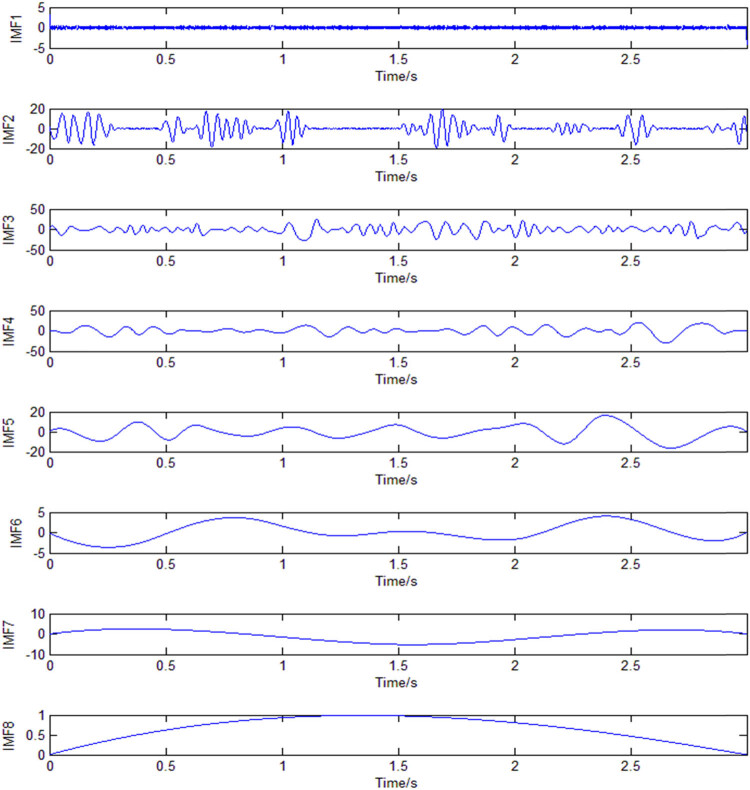
IMF of each order after EMD decomposition of the VMI EEG signal (the vertical axis represents the amplitude in microvolts).

The collected visually imagined EEG data were examined visually, and the data of five subjects with serious EEG contamination were removed. Then, the EEG data of each subject, collected under different tasks, were extracted. The duration of each task was 5 s. To prevent interference of other signals, only the data segments from 1 s (corresponding to 0 s in Figure 3(a)) to 4 s after the task are extracted and analyzed. Figure 3(a) shows the visually imagined original EEG signals of the O1 versus O2 channels during forward and reverse driving of the car.

#### 2.3.2 Preprocessing

First, the baseline drift of the extracted VMI EEG signal is corrected to eliminate the EEG signal deviation from the baseline [13]. According to Kosmyna et al. [15], the visual imaginary EEG signal is related to the alpha band and elliptic filters with 8–13 Hz bandpass. Then, independent component analysis (ICA) [36,37,38,39] removed the electro-ocular artifacts and electromyographic artifacts. Figure 3(c) shows the waveform after the above preprocessing.

#### 2.3.3 HHT-based feature extraction and SVM classification

The HHT method includes empirical mode decomposition (EMD) and Hilbert spectrum analysis (HSA). The EMD obtains the intrinsic mode function (IMF) using the average of the upper and lower envelopes of the time series. The EMD process is as follows: first, all of the maximum and minimum points of the input signal are obtained. Then, the maximum value point and the minimum value point are fitted using a cubic spline. The curve of the upper and lower envelopes is obtained, and the mean value function is calculated to obtain the difference *h* between the analyzed signal and the mean value. Second, we examine whether *h* meets the IMF condition, and if it does, we treat *h* as the first IMF. Otherwise, the first two steps are obtained until the *k*th step satisfies the IMF condition. Then, we obtain the first IMF and calculate the difference *r* between the original signal and the IMF. Finally, the difference *r* is the signal decomposed until the remaining *r* is a monotonic signal or one pole [22]. From the above analysis, the expression of the original signal is shown in equation (1) [22]:

(1) s(t)=\mathop{\sum }\limits_{i=1}^{N}{C}_{i}(t)+{R}_{n}(t),]

where *s*(*t*) is the original signal, *C*_*i*_(*t*) is the IMF component obtained by the *i*th screening, *N* is the number of screening times, and *R*_*n*_(*t*) is the final residual component.

All extractable IMFs are obtained after EMD, and HSA is performed. Equation (2) [22] is a Hilbert spectral transformation for each IMF component:

(2) {Y}_{i}(t)=\frac{1}{\pi }\underset{-\infty }{\overset{+\infty }{\int }}\frac{{C}_{i}(\varphi )}{t-\varphi }\text{d}\varphi .]

The analytical signal of the original signal is as shown in equation (3) [22]:

(3) {X}_{i}(t)={C}_{i}(t)+j{Y}_{i}(t)={A}_{i}(t){e}^{{j}^{p}{i}^{(t)}}.]

The instantaneous amplitude and the instantaneous phase are obtained from equations (4) and (5), respectively [22]:

(4) {A}_{i}(t)=\sqrt{{Y}_{i}{(t)}^{2}+{C}_{i}{(t)}^{2}},]

(5) {P}_{i}(t)=\arctan \frac{{Y}_{i}(t)}{{C}_{i}(t)}.]

The average instantaneous energy is obtained from the instantaneous amplitudes according to equations (6) and (7) [22]:

(6) {\text{EC}}_{N}=\frac{1}{N}\mathop{\sum }\limits_{n=1}^{N}{C}_{n}^{2},\hspace{.25em}N\lt Fs,]

(7) {\text{EC}}_{N}=\frac{1}{N}\mathop{\sum }\limits_{n=N-Fs+1}^{N}{C}_{n}^{2},\hspace{1em}N\gt Fs.]

The left side of equations (6) and (7) is the instantaneous energy value.

After EMD decomposition of the EEG signals of VMI, the IMF of each order is shown in Figure 4. Related studies have shown that [22] the IMF first three orders contribute to classification. The IMF first three orders are combined, and the average instantaneous amplitude is obtained using a Hilbert transform. The sixth-order AR model coefficients AR1–AR6 are extracted using the Burg algorithm. The 12-dimensional feature vector comprises the AR model coefficients of the O1 and O2 channels: {O1_AR1_, …, O1_AR6_, O2_AR1_, …, O2_AR6_}. Then, the SVM suitable for small sample classification is used for feature classification [40]. The classification was carried out using the MATLAB SVM library function and selected with Gauss Kernel Function. A total of 90% of each subject’s data were used to train the SVM model. Thus, 10% of the dataset is used to test the SVM model. A 10-fold cross-validation was implemented to evaluate the experimental performance. Figure 3(d) shows the average classification accuracy of the sub1 visual imaginary car driving forward versus reversing.

## 3 Results

Figure 3 shows the original EEG signal of VMI, the total average waveform contrast of rest and VMI, the waveform after preprocessing, and the waveform after classification. Figure 3(a) shows the original EEG signal of the O1 and O2 channels during visual imagery of forward driving versus reverse driving (a is the original EEG signal of the car driving forward and b is the original EEG signal of the car driving in reverse). Figure 3(b) shows a comparison of the total average waveform of the resting state versus visual imagination. Figure 3(c) shows the waveforms of the O1 and O2 channels undergoing baseline drift correction, 8–13 Hz bandpass filtering, and ICA processing during visual car forward versus reverse driving (where a is the waveform after the process when the car is driven forward and b is the waveform after the process when reversing). Figure 3(d) shows the average classification accuracy of the sub1 visually imagined car traveling forward versus reversing. From Figure 3(b), the EEG of VMI versus rest has two obvious voltage reversals of approximately 200 ms in the position of a and b.

Figure 4 shows the IMF of each order after EMD VMI EEG signal decomposition. As shown in Figure 4, when the waveform is decomposed to the eighth layer, there is only one pole in the waveform, and the decomposition stops. At the same time, the first three orders of IMF contain the required time-frequency information, and the fourth-order IMF contains almost no time-frequency information, consistent with the theoretical analysis in Section 2.3.

Table 1 shows the results of a two-way ANOVA analysis of EEG in each lead under four visual imagery tasks. The mean values of EEG of 25 subjects were calculated in four VMI tasks. The two factors were the type and lead of the visual imagery task. Table 1 shows the mean of visual imagery EEG, revealing that the main effects of electrodes Fp2, F7, P3, O1, Fcz, and Pz are significant (*P* < 0.05), whereas the main effects of other leads are insignificant.

**Table 1:** The results of the EEG analysis of each lead in four VMI tasks using two-way ANOVA (FDR correction)

Leads	df	Mean square	*F*	*P*
Fp2	3	2811.906	6.879	0.000***
F7	3	1153.996	5.078	0.009**
F8	3	700.248	2.784	0.097
F3	3	496.798	2.476	0.118
F4	3	260.193	1.142	0.413
Fc3	3	256.310	1.472	0.330
Fc4	3	390.191	1.932	0.200
C3	3	158.137	1.191	0.413
C4	3	22.740	0.184	0.907
P3	3	1376.669	5.823	0.006**
P4	3	658.243	2.725	0.097
O1	3	903.591	3.485	0.045*
O2	3	86.344	0.386	0.808
Fz	3	216.104	1.108	0.413
Fcz	3	735.923	3.626	0.043*
Cz	3	480.581	2.078	0.182
Pz	3	1518.675	6.385	0.000***
Oz	3	80.270	0.658	0.650

Notes: *P < 0.05, **P < 0.01, and ***P < 0.001.

To verify the VMI movement direction as a mental strategy for BCI users, we carried out the feature extraction and pattern classification of EEG collected during car forward driving and reverse driving of VMI. Table 2 presents the average classification accuracy under different combinations of electrodes. The average classification accuracies of P3 versus P4, O1 versus O2, F7 versus F8, C3 versus C4, F3 versus F4, and Fc3 versus Fc4 were 59.9666 ± 6.7385, 64.7673 ± 4.2748, 53.5205 ± 2.5087, 51.9838 ± 1.8770, 51.5868 ± 1.9974, and 57.6417 ± 5.7230%, respectively. We selected few electrodes for classification as our aim was to explore a less-channel VMI-BCI brain-controlled robotic system. Thus, we carried out only two classifications.

**Table 2:** Average classification accuracy under different electrode combinations

Subjects	Combination of P3 versus P4 (%)	Combination of O1 versus O2 (%)	Combination of F7 versus F8 (%)	Combination of C3 versus C4 (%)	Combination of F3 versus F4 (%)	Combination of Fc3 versus Fc4 (%)
sub1	62.1104	65.1284	55.0210	50.1001	57.0581	51.1177
sub2	53.1567	72.0869	57.0605	51.1284	55.0723	55.1079
sub3	54.1616	62.0781	55.1392	51.1729	50.0830	70.0967
sub4	70.0840	63.1265	51.1157	53.1538	50.0249	53.0591
sub5	69.0127	65.1157	53.0098	55.1880	51.0435	58.0181
sub6	56.0708	64.0430	51.1265	51.2153	50.0835	70.0620
sub7	58.1177	66.0664	52.1313	50.1846	50.1572	64.0684
sub8	63.0933	65.0854	53.1152	50.1709	51.1211	62.0034
sub9	53.1284	65.0898	52.0215	51.1553	50.1104	55.0767
sub10	52.1074	63.1245	63.0771	51.1055	52.0137	54.1226
sub11	53.1006	59.1787	55.1221	52.0249	56.0103	55.1484
sub12	65.0493	62.1885	52.1377	51.0049	52.0693	63.1265
sub13	54.0244	73.1714	53.0918	53.0391	51.0444	52.0244
sub14	54.1465	55.1118	54.1040	51.0049	50.0439	58.1138
sub15	68.1597	69.0474	51.1392	54.1064	50.0381	54.1489
sub16	69.1919	72.0327	51.0703	52.1079	50.0918	62.0884
sub17	58.0954	68.0562	53.0278	51.1113	51.1182	66.0752
sub18	52.0278	65.1812	52.0249	53.1587	53.1245	51.0562
sub19	69.0059	67.2466	52.1025	51.1665	54.1011	53.0142
sub20	58.0527	62.2261	53.1382	51.0933	51.1265	55.0322
sub21	57.0342	61.1816	52.1436	51.0044	50.0776	62.1289
sub22	62.0791	69.1982	54.0620	57.0522	50.0254	54.1582
sub23	52.0586	59.1909	55.0054	56.0874	51.0010	51.0806
sub24	72.0166	62.1611	54.0093	51.0527	52.0171	56.0625
sub25	64.0815	63.0669	53.0176	50.0073	51.0151	55.0532
Average classification rate	59.9666	64.7673	53.5205	51.9838	51.5868	57.6417
Variance	6.7385	4.2748	2.5087	1.8770	1.9974	5.7230

Table 3 shows the descriptive statistical results of EEG in each lead in four VMI tasks. Table 4 shows the results of principal effect analysis using a two-way ANOVA of different VMI tasks in Table 3. Table 4 shows the different leads between groups, which visually imagined the car moving forward, reversing, turning left, and turning right. Table 4 showed that the differences between groups of the Fp2, F7, P3, O1, Fcz, and Pz leads, revealing that they were significant (*P* < 0.05).

**Table 3:** The EEG descriptive statistical results in each lead during the four visual imagery tasks, with the standard deviation in parentheses and the mean value outside parentheses

Leads	Visual imagination driving forward	Visual imagination reversing	Visual imagination turning left	Visual imagination turning right
Fp2	0.150 (26.75)	0.789 (14.09)	0.529 (19.02)	0.804 (18.94)
Pz	0.529 (16.08)	0.729 (14.84)	0.338 (14.95)	0.850 (15.76)
P3	0.258 (17.80)	0.657 (12.61)	0.405 (15.37)	0.713 (15.27)
F7	0.463 (16.63)	0.738 (13.53)	0.456 (14.83)	0.844 (15.13)
Fcz	0.453 (15.18)	0.704 (13.38)	0.455 (13.94)	0.744 (14.40)
O1	0.460 (17.81)	0.739 (14.50)	0.563 (15.82)	0.847 (16.09)

**Table 4:** Results of the main effect analysis of four visual imagery tasks under different leads (FDR correction)

Leads	Source	Sum of squares	Mean square	*F*	*P*
Fp2	Between groups	1813.333	5.965	4.818	0.000
	Intergroup	148186.667	1.238		
	Total	150000.000			
Pz	Between groups	422.081	2.833	2.270	0.000
	Intergroup	149577.919	1.248		
	Total	150000.000			
P3	Between groups	800.732	3.906	3.136	0.006
	Intergroup	149199.268	1.245		
	Total	150000.000			
F7	Between groups	662.188	3.638	2.919	0.009
	Intergroup	149337.812	1.246		
	Total	150000.000			
Fcz	Between groups	453.109	3.001	2.405	0.043
	Intergroup	149546.891	1.248		
	Total	150000.000			
O1	Between groups	671.761	3.499	2.807	0.045
	Intergroup	149328.239	1.246		
	Total	150000.000			

Table 5 presents an average classification of 25 subjects visually imagining the car’s forward versus reverse, forward versus left, forward versus right, reverse versus left, reverse versus right, and left versus right movements. The average classification accuracies are 64.76 ± 4.27, 56.49 ± 4.63, 55.78 ± 3.09, 61.11 ± 4.56, 64.30 ± 7.13, and 73.66 ± 6.80%, respectively. The electrode combination is O1 versus O2.

**Table 5:** Average classification accuracy of different visual imagery task pairs for 25 subjects under electrode combinations O1 and O2

Subjects	Visual imagination driving forward versus reversing (%)	Visual imagination driving forward versus turning left (%)	Visual imagination driving forward versus turning right (%)	Visual imagination reversing versus turning left (%)	Visual imagination reversing versus turning right (%)	Visual imagination left turn versus right turn (%)
sub1	65.1284	59.1079	60.0303	62.0361	63.0684	73.0737
sub2	72.0869	57.1860	55.0093	58.0269	58.1099	78.1523
sub3	62.0781	58.2417	52.0151	65.1533	74.0347	82.0259
sub4	63.1265	68.2012	54.0254	56.0220	56.0742	63.0039
sub5	65.1157	57.1509	53.0610	66.0933	72.0630	59.1274
sub6	64.0430	68.0151	55.0869	63.0981	65.1328	79.0356
sub7	66.0664	57.0659	56.0313	59.0737	73.0708	68.0308
sub8	65.0854	55.0449	58.0269	63.0815	66.0117	63.0020
sub9	65.0898	55.0352	58.1108	55.0010	64.0283	76.1270
sub10	63.1245	56.0083	61.0962	57.0933	71.0986	75.1089
sub11	59.1787	50.0376	55.1216	65.0200	51.0210	75.0151
sub12	62.1885	58.1094	60.0410	56.0229	50.0469	67.0474
sub13	73.1714	58.1250	53.0293	60.0015	59.0454	68.0166
sub14	55.1118	57.0879	54.0010	68.1064	73.0327	82.0073
sub15	69.0474	58.1299	56.1240	65.0444	65.0132	79.0547
sub16	72.0327	59.2061	53.0229	54.0161	66.0156	73.0576
sub17	68.0562	51.0029	55.0381	59.1025	72.0962	67.0610
sub18	65.1812	50.0518	56.1567	73.1294	59.0869	68.1665
sub19	67.2466	50.1187	52.1240	65.0840	55.0747	75.1328
sub20	62.2261	59.0928	59.0669	62.0684	67.1313	85.0654
sub21	61.1816	57.0010	52.0444	57.0947	59.0229	77.0098
sub22	69.1982	55.1304	61.0200	59.0205	73.0957	76.0820
sub23	59.1909	55.1714	56.0601	62.1230	61.1050	83.0894
sub24	62.1611	52.0532	59.1353	59.1401	71.1045	71.1309
sub25	63.0669	51.0503	50.1255	58.1221	63.0928	78.0713
Average classification rate	64.7673	56.4970	55.7841	61.1110	64.3070	73.6678
Variance	4.2748	4.6322	3.0904	4.5623	7.1310	6.8068

**Figure 5:**
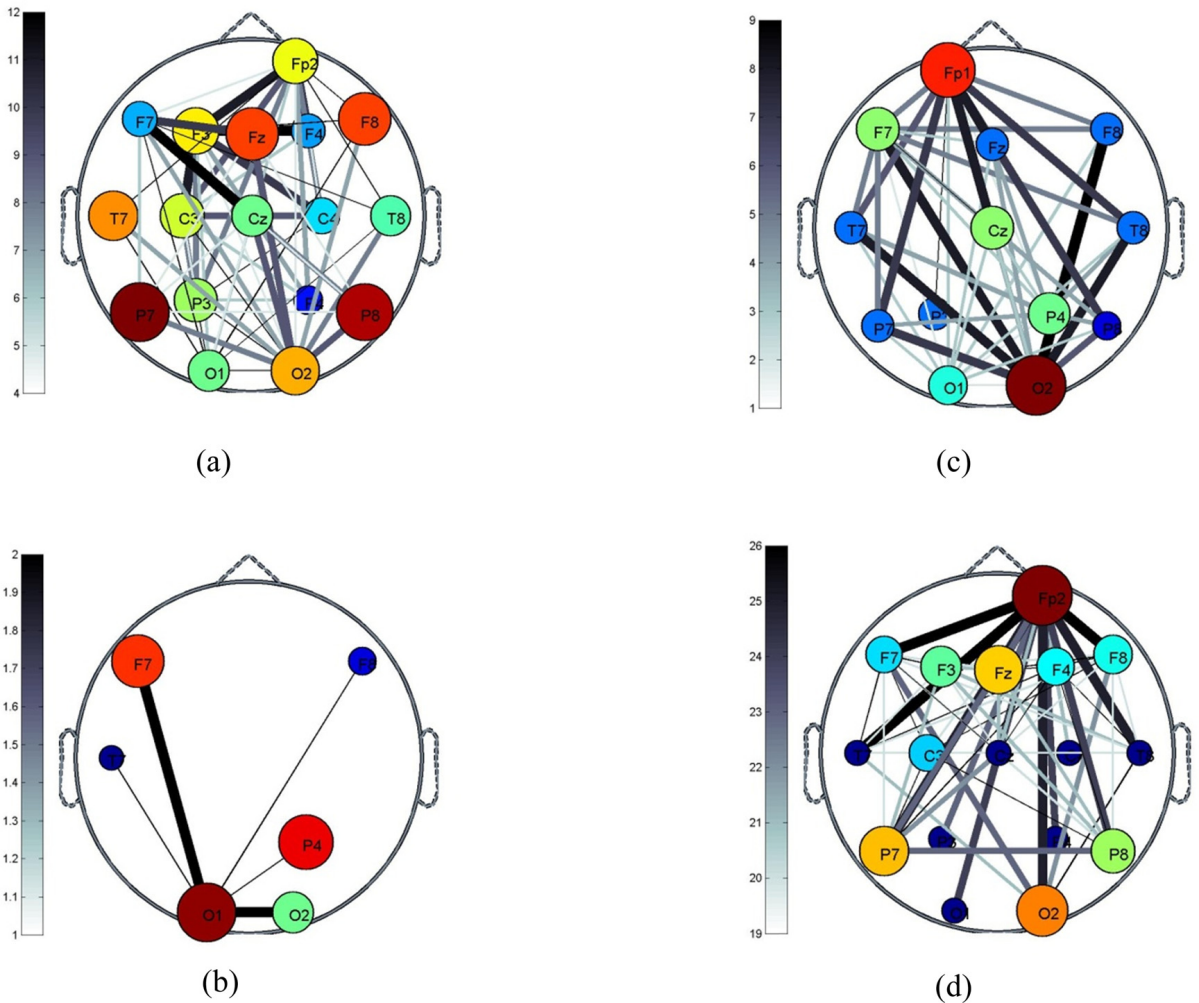
The correlation of 18 channels of EEG during VMI: (a) the correlation of the lead when the car moves forward in the visual imagination; (b) the correlation of the lead when the car reverses; (c) the correlation of the lead when the car turns left; and (d) the correlation of the lead when the car turns right.

Figure 5 presents the correlation of 18 channels of EEG during VMI to examine the correlation between the signals of the channel in relevant brain regions during visual imagery. The thickness and color of the connection between the two nodes show the degree of their correlation. Figure 5 shows that O1 and F7 have the strongest connectivity during the car reversal period of the visual imagination. In the car left turn, the connection between O2 and F8 is the strongest. In the car right turns, the connection between O2 and Fp2 is the strongest. Thus, Cz and F7 have the strongest connectivity when the car is moving forward.

## 4 Discussion

Many earlier studies have made a remarkable research progress on MI-BCI [15,16,17]; however, research on VMI-BCI is slow. The design of BCI’s paradigm and feature extraction needs to be further explored. This article focuses on the VMI-BCI paradigm and feature extraction. At present, few studies have explored predefined VMI (guided by prompting visual observation). A research on this VMI is challenging, and it is necessary to design an appropriate experimental paradigm to evaluate the visual imagination ability of subjects and identify different visual imagination tasks. In this study, a new VMI-BCI paradigm was designed, in which subjects were asked to complete four VMI tasks: imagine a car moving forward, reversing, turning left, and turning right, and then, we use HHT to extract features and classify them.

The experimental paradigm is different from that of Nataliya et al. [15], which requires subjects to visually imagine static flowers versus hammers, whereas this study requires subjects to visually imagine dynamic cars. Sousa [20] showed that dynamic visual imagination induces stronger brain activation than static visual imagination. The classification results of the different visual imagery tasks designed in this study (the average classification accuracy of the left versus right turns of the visual imagination car is 73.66 ± 6.80%) indicate that the experimental paradigm has good separability. Table 4 indicates that the visually imaginary car can be distinguished from forward, backward, left, and right turns by selecting the appropriate lead to extract features. This shows that the experimental paradigm of visual imagination is feasible. Second, the experimental paradigm is different from other experimental paradigms [15,18,19,20] based on the visual imagination of the same object (car), but the direction of movement is different. This design explores the difference of visual imagination in controlling direction variables. However, this design poses a challenge to feature extraction and classification. Better methods are needed to obtain classification results. In contrast, different driving directions of the VMI car can naturally correspond with the forward, backward, left, and right turn of a control robot or a car, which may provide the practical application of visual-motor-imagery-based brain-controlled robot or car.

The EEG pattern induced by the VMI task is also closely related to the performance of subjects’ imaginative mental activities [4]. To this end, 25 healthy volunteers with strong VMI ability (questionnaire score ≥70) were recruited before the experiment according to the VMIQ [24,25,26,27,28,29,30,32] or the KVMIQ [23,31,33,34,35]. After the experiment, each participant was asked to fill in a questionnaire of the visual imagery car movement direction task. Compared with performing MI tasks, most subjects consider VMI tasks to be a mental task, which is easier to accomplish. Although all subjects did not perform specialized VMI training, they can complete the VMI task better.

For the feature extraction of VMI-BCI, the EEG power spectrum estimation method is mainly used in the existing research [15,18,19,20,41,42], whereas the HHT method, which can obtain high-resolution information in both the time domain and the frequency domain, is used to extract features in this article. Thus, SVM was used to classify the forward and reverse, forward and left turn, forward and right turn, reverse and left turn, reverse and right turn, and left and right turn. Among them, the highest average classification accuracy is 73.66 ± 6.80%, and the average classification accuracy is 62.68 ± 5.08%. The average classification accuracy of this study is 6.68% points higher than that of Neuper et al. [18], in which the VMI is 56%. Compared with Kosmyna et al. [15], the average classification accuracy of the visual imagination flower and the visual imagination hammer is 52%, improved by 10.68 percentage points. This indicates that the features of VMI-EEG extracted from HHT have certain separability for VMI tasks.

When extracting the EEG features of VMI using HHT, the selection and combination of electrodes have an impact on the classification accuracy of VMI tasks. Table 1 indicates that the EEG mean values of the electrodes Fp2, F7, P3, O1, Fcz, and Pz are different from the designed VMI task. Figure 5 illustrates the strongest connection between the electrode pairs O1 and F7, O2 and F8, O2 and Fp2, and Cz and F7 during visual imaginary car reversing, left turn, right turn, and forward travel. The classification results in Table 2 showed that the classification accuracy of the combination of O1 versus O2 electrodes was the highest. The two-way ANOVA in Tables 3 and 4 showed that the interaction between the O1 and O2 electrodes was significant during the VMI (*P* = 0.045, *P* < 0.05). The first reason for these results is that VMI is related to memory, whereas memory is related to the prefrontal cortex and the endothelial layer of the lateral interior of the parietal lobe (LIP area) [4,7]. The electrodes Fp2, F7, Fcz, P3, and Pz are located in these cortex areas. There are two visual information processing pathways: one is from the dorsal striate cortex to the parietal lobe, which mainly analyzes motion vision, and the second is from the ventral projection to the temporal lobe that mainly recognizes objects [4]. Therefore, the occipital region participates in the neuroprocessing of the VMI and can analyze the EEG features of the O1 versus O2 electrodes to identify the VMI task.

The EEG features of electrodes Fp2, F7, Fcz, P3, and Pz in VMI have been analyzed, and the correlation between channel EEG and VMI [4,5,6,7,8,9,10,15,19,20] has been calculated. However, the EEG features of O1 and O2 electrodes in VMI have not been analyzed and extracted. The EEG features of O1 versus O2 electrodes in VMI were analyzed and extracted using HHT. The classification results in Table 5 showed that the designed VMI tasks can be distinguished.

Compared with the traditional MI-BCI with more research, the task based on EEG recognition VMI is more challenging. The research is still insufficient, and the classification accuracy needs to be further improved. For this reason, feature selection and extraction and electrode combination optimization should be studied.

In our future work, we will attempt to extract the correlation between channel EEGs in VMI as a feature to optimize the combination of electrodes and to improve the classification accuracy of VMI tasks. We will construct an online real-time VMI-BCI brain-controlled robot system with few channel EEGs.

## 5 Conclusion

Contrary to the traditional MI-BCI paradigm, this study designed a new VMI-BCI paradigm: visual imagination of a car moving forward, reversing, turning left, and turning right. These four mental strategies can control the car or robot to move forward, backward, left, and right. EEG features are extracted from HHT with high temporal and spatial resolution, and the designed VMI task is identified using an SVM suitable for small sample classification. Studies have shown that in the designed experimental paradigm, the above EEG extracted features at the O1 versus O2 electrode positions in the occipital region can distinguish visual imagination tasks (average classification accuracy can reach 73.66 ± 6.80%). Visual imagination car has the strongest connection between the electrode pairs O1 and F7, O2 and F8, O2 and Fp2, and Cz and F7 in reversing, turning left, turning right, and driving forward. The visual imagination driving car based on EEG is expected to be a BCI strategy, and VMI-BCI can be used as a window to observe brain function. This article can provide ideas for online real-time VMI-BCI brain-controlled robot systems based on less-channel EEG.
